# 

**Published:** 1861-10

**Authors:** 


### POSTPONEMENT.

The session of the Ohio College of Dental Surgery, for the coming winter has been postponed. At a recent meeting of the faculty, it was thought best, after duly considering the subject, to pursue that course. The upbroken condition in which all things are, seem to warrant the conclusion, that the class would be very small. The members of the faculty expressed a willingness to devote their time, but not time and money both, to the institution, especially in such times of pecuniary disaster as the present. T.

### DENTAL REGISTER,

### OF THE WEST.

### Issued Monthly, at $3 a year in advance.

DEVOTED TO THE INTERESTS OF THE DENTAL PROFESSION.

### Edited by J. TAFT and GEO. WATT.

The Volume commences in January and closes in December.

The Register being issued monthly is always fresh, therefore indispensable to the practitioner who desires to keep posted up with the new inventions and improvements which are daily developed. Neither expense nor effort will be spared to give value and variety to its contents. Articles will be illustrated with well executed engravings, whenever they will add to the interest or assist in explanation.

Its extensive circulation and frequency of issue makes it the BEST DENTAL ADVERTISING MEDIUM in the United States.

☞ CONTRIBUTIONS SOLICITED.

Address Original Communications to **Geo. Watt, Xenia, O.**

Exchanges to **J. Taft**, or **Dental Register, Cincinnati, O.**

Business Communications, to

**JNO. T. TOLAND, Publisher and Proprietor,**
38 West Fourth Street, Cincinnati, O.

Communications must reach the Editor as early as the 15th, to insure insertion in the next issue.

